# Design and Synthesis of New 2-Aryl-4,5-dihydro-thiazole Analogues: *In Vitro* Antibacterial Activities and Preliminary Mechanism of Action

**DOI:** 10.3390/molecules201119680

**Published:** 2015-11-09

**Authors:** Fangfang Tan, Baojun Shi, Jian Li, Wenjun Wu, Jiwen Zhang

**Affiliations:** 1 College of Science, Northwest Agriculture and Forestry University, Yangling 712100, China; 15877653513@163.com (F.T.); lijian3773@163.com (J.L.); 2 Institute of Pesticide Science, Northwest Agriculture and Forestry University, Yangling 712100, China; shibaojun@nwsuaf.edu.cn (B.S.); wuwenjun@nwsuaf.edu.cn (W.W.)

**Keywords:** synthesis, 4,5-dihydrothiazole, antibacterial activity, antibacterial mechanism

## Abstract

Sixty 2-aryl-4,5-dihydrothiazoles were designed and synthesized in yields ranging from 64% to 89% from cysteine and substituted-benzonitriles via a novel metal- and catalyst-free method. The structures of the title compounds were confirmed mainly by NMR spectral data analysis. Antibacterial activity assays showed that the compounds (*S*)-2-(2′-hydroxyphenyl)-4-hydroxy-methyl-4,5-dihydrothiazole (**7h**) and (*R*)-2-(2′-hydroxyphenyl)-4-hydroxymethyl-4,5-dihydro-thiazole (**7h**′) exhibited significant inhibition against *Ralstonia solanacearum*, *Pseudomonas syringae* pv. *actinidiae*, *Bacillus subtilis* and *Bacillus cereus*, with minimum inhibitory concentrations (MICs) ranging from 3.91 to 31.24 μg·mL^−1^. The effect of substituents showed that not only electron-withdrawing groups, but also electron-donating groups could abolish the antibacterial activities unless a 2′-hydroxy group was introduced on the 2-aryl substituent of the 4,5-dihydrothiazole analogues. The results of scanning electron microscope (SEM) and fatty acid exposure experiments indicated that these antibacterial compounds influence fatty acid synthesis in the tested bacteria.

## 1. Introduction

*Ralstonia solanacearum* and *Pseudomonas syringae* pv. *Actinidiae* are the causes of serious bacterial diseases that pose a major threat to agricultural production worldwide [1]. Unfortunately, there have been few reports of effective bactericides against these pathogens. In the process of screening new agricultural bactericides, we found that some 2-arylthiazolines such as (*S*)-2-(2′-hydroxyphenyl)-4-hydroxymethyl-4,5-dihydrothiazole and (*R*)-2-(2′-hydroxyphenyl)-4-hydroxymethyl-4,5-dihydrothiazole exhibited potent antibacterial activities [2,3,4,5]. Inspired by these results, we wondered whether the 2-arylthiazoline scaffold could be a lead compound for agricultural bactericides. In order to investigate the bactericidal activities and clarify the inevitable structural motifs of 2-arylthiazoline analogues, sixty 2-aryl-4,5-dihydrothiazoles were then designed and synthesized.

A couple of protocols have been developed for the synthesis of 2-arylthiazoline scaffolds, which mainly include using ethyl benzimidates reacted with cysteine [6], aryl nitriles condensed with cysteine in buffered media [7], Ru-catalyzed/TBHP oxidation reactions [8], and so on [9,10,11]. The broad application of these methods has been hindered by the following drawbacks: long reaction time, high reaction temperatures (>100 °C), tedious work-ups and the use of expensive noble metal catalysts.

**Figure 1:**
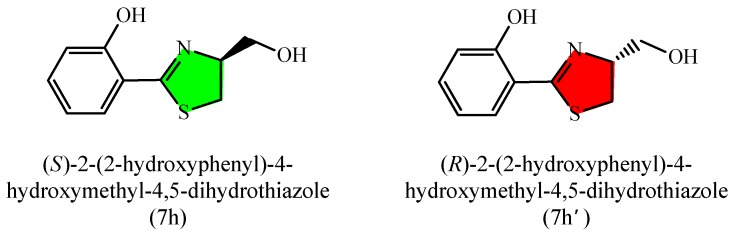
Chemical structures of (*S*)-2-(2-hydroxyphenyl)-4-hydroxymethyl-4,5-dihydrothiazole and (*R*)-2-(2-hydroxyphenyl)-4-hydroxymethyl-4,5-dihydrothiazole.

We present herein the design and synthesis a series of 2-aryl-4,5-dihydrothiazolines under mild metal- and catalyst-free conditions [12] as well as their *in vitro* antibacterial activities against *Ralstonia solanacearum* (*R. solanacearum*), *Pseudomonas syringae* pv. *Actinidiae* (*P. syringae*), *Bacillus subtilis* (*B. subtilis*) and *Bacillus cereus* (*B. cereus*). Furthermore, we also explore the preliminary antibacterial mechanism of compound **7h** (Figure 1).

## 2. Results and Discussion

### 2.1. Preparation of 2-Arylthiazoline Analogues

**Scheme 1:**
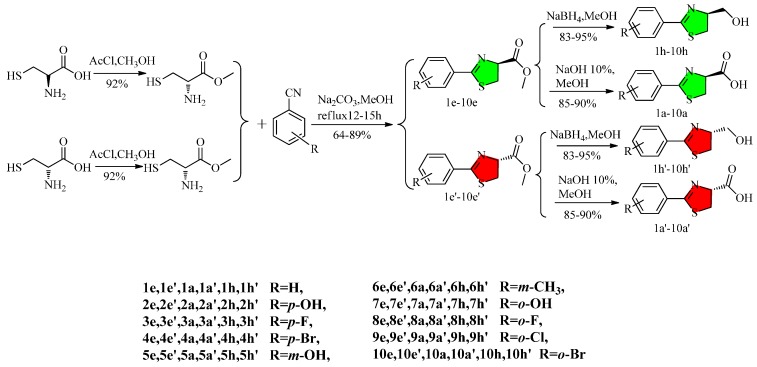
Synthetic route of 2-arylthiazoline analogues.

The synthetic route to the 2-arylthiazoline analogues is depicted in Scheme 1. Aryl nitriles and methyl cysteine were used as starting materials and reacted in dry methanol in the presence of sodium carbonate and refluxed for 12–15 h to obtain methyl-2-aryl-4,5-dihydrothiazole-4-carboxylates in yields ranging from 64% to 89% [12]. 

The results suggested that *ortho*-substituents did not favor the reaction, in particular larger groups, such as -Br and -Cl. Meanwhile the electron-withdrawing groups were better in this reaction than electron-donating ones. The carboxylates **1e**–**10e**, **1e′**–**10e′** were reduced by NaBH_4_ in EtOH to furnish the corresponding 2-aryl-4-hydroxymethyly-4,5-dihydrothiazoles **1h**–**10h**, **1h′**–**10h′** and treated with 10% sodium hydroxide solution in EtOH to get the corresponding 2-aryl-4,5-dihydrothiazole-4-carboxylic acids **1a**–**10a**, **1a′**–**10a′**. The structures of all the compounds were confirmed mainly by analyses of their NMR spectral and mass spectrometry data or reference to related literature [3,13,14,15].

### 2.2. Antibacterial Activity Assays

All compounds were evaluated for *in vitro* antibacterial activities against two Gram-positive bacteria—*B. cereus* and *B. subtilis*—and two Gram-negative bacteria—*R. solanacearum* and *P. syringae*. The inhibition zone diameters and minimum inhibitory concentrations (MICs), presented in Table 1 and Table 2, were determined by the filter paper and double-dilution methods, respectively. However, only six 2′-hydroxyphenyl-4,5-dihydro-thiazoles showed potent antibacterial activities at the tested concentration, in particular, compounds **7h** and **7h′** showed the highest antibacterial activities for all tested bacteria.

**Table 1:** Inhibition Zone Diameters of Six Compounds against the Tested Bacteria.

Compounds	Diameter of Inhibition Zone (mm) in 10 μL/Disk (Mean ± S.D.)			
	*B. subtilis*	*B. cereus*	*R. solanacearum*	*P. syringae*
**7a**	-	-	-	11.0 ± 0.23 (+)
**7a′**	-	-	-	10.5 ± 0.56 (+)
**7e**	12.3 ± 0.02 (+)	-	9.1 ± 0.11 (++)	-
**7e′**	11.2 ± 0.45 (+)	-	9.6 ± 0.43 (++)	-
**7h**	20.5 ± 0.56 (+++)	16.3 ± 0.23 (+++)	15.5 ± 0.13 (++)	18.2 ± 0.34 (+++)
**7h′**	20.0 ± 0.22 (+++)	16.7 ± 0.11 (+++)	14.9 ± 0.55 (++)	17.8 ± 0.23 (+++)

All values were means of three replicates, “+++” means transparent; “++” means clear; “+” means visible; “−” means no inhibitory effect.

**Table 2:** The MICs of Four Compounds against the Tested Bacteria.

Tested Bacteria	Minimum Inhibitory Concentration (μg·mL^−1^)				
	7h	7h′	7e	7e′	Ampicillin
*R. solanacearum*	31.24	31.24	>125	>125	>125
*P. syringae*	7.81	7.81	>125	>125	>125
*B. cereus*	31.24	31.24	>125	>125	62.5
*B. subtilis*	3.91	3.91	>125	>125	>125

### 2.3. Mechanism of Antibacterial Action

#### 2.3.1. Fatty Acid Exposure Experiment

**Figure 2:**
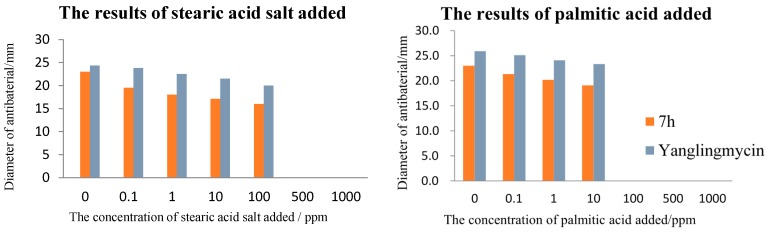

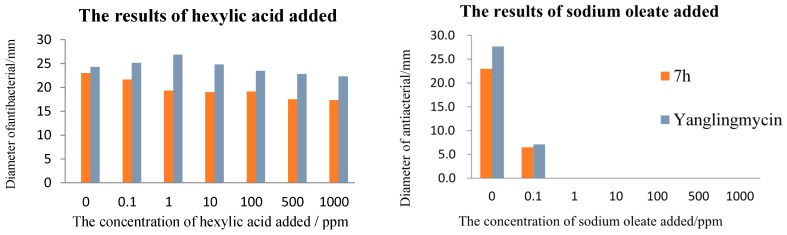
The fatty acid exposure experiments of compound **7h**.

The results of the fatty acid exposure experiments was similar to those of yaglingmycin, that is the diameters of inhibition zone decrease significantly with the increase of the concentration of some fatty acids. It was worth nothing that compound **7h** completely lost the antibacterial activity against *B. subtilis* in the presence of the long-chain saturated stearic acid, palmitic acid, and long-chain unsaturated oleic acid at the concentration of 500 ppm, 100 ppm, 1 ppm, respectively. Nevertheless, compound **7h** did not lose the antibacterial activity against *B. subtilis* in the presence of short-chain *n*-hexylic acid and these results can be found in Figure 2.

#### 2.3.2. Scanning Electron Microscope (SEM)

**Figure 3:**
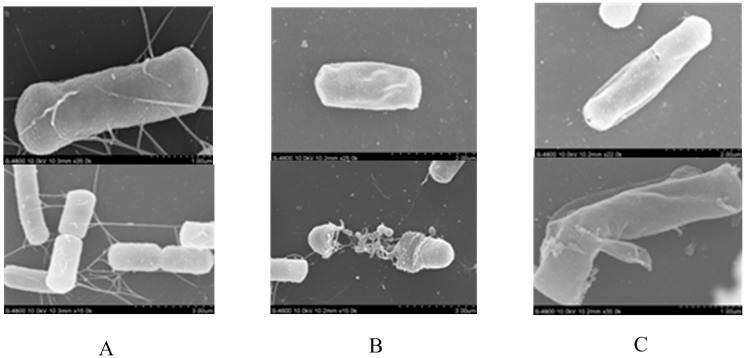
Scanning Electron Microscope (SEM) photography of *B. cereus.* (**A**) Blank control; (**B**) Positive control (treated with yanglingmycin); (**C**) Treated with Compound **7h**.

After treating *B. cereus* with 31.24 μg·mL^−1^ **7h** for 3 h, the morphological changes to the bacterial cells were observed by Scanning Electron Microscope (SEM). As shown in Figure 3, the surfaces of untreated bacterial cells were smooth and intact, while the morphology of treated cells changed dramatically. The treated cells had obvious depressions and deformations, some clear holes in the damaged cell walls and cell membranes and the cells became irregular, pitted, and shriveled. The morphological changes on the surface might result in diffusion of the contents from the cells to the outside.

## 3. Experimental Section

### 3.1. General Information

Solvents were of analytical reagent (AR) grade unless otherwise mentioned. TLC was performed on E. Merck 60 F254 silica gel plates. Column chromatography was carried out with 200~300 mesh silica gel (Qingdao Haiyang Co., Ltd., Qingdao, Shandong, China); compounds were eluted with the mixture solution of petroleum ether and ethyl acetate in sequence. Melting points (m.p.s, uncorrected) were measured using a Yanagimoto apparatus (Shanghai-Measuring Equipment Co., Ltd., Shanghai, China). ^1^H-NMR (500 MHz) and ^13^C-NMR (125 MHz) were obtained on a Bruker-Avance-500 spectrometer (Bruker Corporation, Switzerland) with DMSO-*d*_6_ or CDCl_3_ as solvent and SiMe_4_ as internal standard. Mass spectra were recorded under electrospray ionization (ESI) conditions by using a Thermo LCQ Fleet instrument (Thermo Fisher, Waltham, MA, USA).

### 3.2. Chemistry

#### 3.2.1. Synthesis of **1e**–**10e** and **1e′**–**10e′**

Acetyl chloride (1.6 mL, 22 mmol) was slowly added to anhydrous MeOH (100 mL) at 0 °C. The solution was then stirred for 5 min, followed by addition of an amino acid (d-cysteine or l-cysteine) (20 mmol). After refluxing for 6 h, the reaction mixture was cooled to room temperature, and evaporated under reduced pressure to furnish the methyl d/l-cysteinate as a white solid in yields of 90% or 85%, respectively. To the anhydrous methanol solution (1.0 mL) of the benzonitriles (1.0 mmol) and anhydrous Na_2_CO_3_ (106 mg, 1.0 mmol) the above synthesized methyl cysteinate (5.0 mmol) was added, then the resulting mixture was stirred at 80 °C for 12 h, cooled to room temperature and concentrated. The crude mixture was purified by column chromatography on silica gel (PE/EA) to afford the desired 2-substituted-phenyl-4-methoxycarbonyl-4,5-dihydrothiazoles **1e**–**10e** and **1e′**–**10e′** in 76% to 85% yields.

*(S)-2-Phenyl-4-methoxycarbonyl-4,5-dihydrothiazole* (**1e**). White powder. m.p. 60–62 °C. ^1^H-NMR (CDCl_3_) δ 7.87 (d, *J =* 7.6 Hz, 2H), 7.50–7.40 (m, 3H), 5.30 (t, *J =* 9.1 Hz, 1H), 3.84 (s, 3 H), 3.73 (dd, *J* = 8.8, 11.0 Hz, 1H), 3.67–3.62 (m, 1H). ^13^C-NMR (CDCl_3_) δ 171.3, 171.0, 132.6, 131.7, 128.6 × 2, 128.5 × 2, 78.5, 52.7, 35.3. ESI-MS *m*/*z*: Calcd for C_11_H_11_NO_2_S: 222.05 [M + H]^+^; found: 222.35. ${\left[\alpha \right]}_{\text{D}}^{20}$ +129.0° (*c* = 0.1, MeOH).

*(S)-2-(4′-Hydroxyphenyl)-4-methoxycarbonyl-4,5-dihydrothiazole* (**2e**). White powder. m.p. 166–168 °C. ^1^H-NMR (CDCl_3_) δ 7.69 (d, *J =* 8.5 Hz, 2H), 6.76 (d, *J =* 8.5 Hz, 2H), 5.28 (t, *J =* 8.8 Hz, 1H), 3.79–3.77 (m, 3H), 3.72–3.62 (m, 2H). ^13^C-NMR (CDCl_3_) δ 172.2, 171.5, 159.8, 130.6 × 2, 124.4, 115.6 × 2, 77.6, 52.8, 35.3. ESI-MS *m*/*z*: Calcd for C_11_H_11_NO_3_S: 238.05 [M + H]^+^; found: 238.26. ${\left[\alpha \right]}_{\text{D}}^{20}$ +109.6° (*c* = 0.1, MeOH).

*(S)-2-(4′-Fluorophenyl)-4-methoxycarbonyl-4,5-dihydrothiazole* (**3e**). Yellow oil. ^1^H-NMR (CDCl_3_) δ 7.89–7.86 (m, 2 H), 7.10 (t, *J =* 8.7 Hz, 2H), 5.28 (t, *J =* 9.1 Hz, 1H), 3.85 (s, 3H), 3.76–3.64 (m, 2H). ^13^C-NMR (CDCl_3_) δ 171.2, 169.7, 164.8 (d, *J =* 252 Hz), 130.8, 130.7, 129.0, 115.7, 115.5, 78.4, 52.8, 35.7. ESI-MS *m*/*z*: Calcd for C_11_H_10_FNO_2_S: 240.04 [M + H]^+^; found: 240.18. ${\left[\alpha \right]}_{\text{D}}^{20}$ +57.8° (*c* = 0.1, MeOH).

*(S)-2-(4′-Bromophenyl)-4-methoxycarbonyl-4,5-dihydrothiazole* (**4e**). White solid. m.p. 74–76 °C. ^1^H-NMR (CDCl_3_) δ 7.74–7.73 (m, 2H), 7.56–7.54 (m, 2H), 5.27 (t, *J =* 9.1 Hz, 1H), 3.84 (s, 3H), 3.74 (dd, *J =* 8.8, 11.0 Hz, 1H), 3.66 (dd, *J =* 9.5, 11.0 Hz, 1H). ^13^C-NMR (CDCl_3_) δ 171.1, 169.9, 131.7 × 2, 131.5, 130.0 × 2, 126.3, 78.5, 52.8, 35.6. ESI-MS *m*/*z*: Calcd for C_11_H_10_BrNO_2_S: 299.96:301.96 = 1:1 [M + H]^+^; found: 299.69:301.85 = 1:1. ${\left[\alpha \right]}_{\text{D}}^{20}$ +7.6° (*c* = 0.1, MeOH).

*(S)-2-(3′-Hydroxyphenyl)-4-methoxycarbonyl-4,5-dihydrothiazole* (**5e**). Colourless oil. ^1^H-NMR (CDCl_3_) δ 7.37 (s, 1H), 7.32 (d, *J =* 7.6 Hz, 1H), 7.22 (t, *J =* 8.0 Hz, 1H), 6.96 (dd, *J =* 1.9, 8.2 Hz, 1H), 5.29 (t, *J =* 9.1 Hz, 1H), 3.80 (s, 3H), 3.72–3.62 (m, 2H). ^13^C-NMR (CDCl_3_) δ 172.2, 171.3, 156.2, 133.4, 129.8, 120.9, 119.4, 114.9, 77.9, 52.8, 35.2. ESI-MS *m*/*z*: Calcd for C_11_H_11_NO_3_S: 238.05 [M + H]^+^; found: 238.22. ${\left[\alpha \right]}_{\text{D}}^{20}$ +50.2° (*c* = 0.1, MeOH).

*(S)-2-(3′-Methylphenyl)-4-methoxycarbonyl-4,5-dihydrothiazole* (**6e**). Colourless oil. ^1^H-NMR (CDCl_3_) δ 7.72 (s, 1H), 7.64 (d, *J =* 4.1 Hz, 1H), 7.30 (d, *J =* 4.4 Hz, 2H), 5.29 (t, *J =* 9.1 Hz, 1H), 3.84 (s, 3H), 3.73–3.62 (m, 2H), 2.39 (s, 3H). ^13^C-NMR (CDCl_3_) δ 171.4, 171.2, 138.3, 132.6, 132.5, 129.0, 128.4, 125.9, 78.4, 52.8, 35.3, 21.2. ESI-MS *m*/*z*: Calcd for C_12_H_13_NO_2_S: 236.07 [M + H]^+^; found: 236.41. ${\left[\alpha \right]}_{\text{D}}^{20}$ +71.7° (*c* = 0.1, MeOH).

*(S)-2-(2′-Hydroxyphenyl)-4-methoxycarbonyl-4,5-dihydrothiazole* (**7e**). Colorless oil. ${\left[\alpha \right]}_{\text{D}}^{20}$ +13.5° (*c* = 0.1, MeOH) [3].

*(S)-2-(2′-Fluorophenyl)-4-methoxycarbonyl-4,5-dihydrothiazole* (**8e**). White solid. m.p. 70–72 °C. ^1^H-NMR (CDCl_3_) δ 7.95–7.92 (m, 1H), 7.47–7.43 (m, 1H), 7.21–7.11 (m, 2H), 5.26 (t, *J =* 9.5 Hz, 1H), 3.84 (s, 3H), 3.74–3.62 (m, 2H) ^13^C-NMR (CDCl_3_) δ 171.2, 166.0 (d, *J =* 5.04 Hz), 160.6, (d, *J =* 255.78 Hz), 133.0 (d, *J =* 10.89 Hz), 130.7(d, *J =* 2.52 Hz), 124.1(d, *J =* 3.78 Hz), 120.7(d, *J* = 11.43 Hz), 116.3(d, *J =* 22.68 Hz), 52.8, 40.1, 35.4. ESI-MS *m*/*z*: Calcd for C_11_H_10_FNO_2_S: 240.04 [M + H]^+^; found: 240.12. ${\left[\alpha \right]}_{\text{D}}^{20}$ +14.4° (*c* = 0.1, MeOH).

*(S)-2-(2′-Chlorophenyl)-4-methoxycarbonyl-4,5-dihydrothiazole* (**9e**). Yellow oil. ^1^H-NMR (CDCl_3_) δ 7.66 (d, *J =* 7.6 Hz, 1H), 7.44–7.42 (m, 1H), 7.37–7.28 (m, 2H), 5.31 (t, *J =* 9.5 Hz, 1H), 3.85 (s, 3H), 3.79 (dd, *J =* 9.5, 11.0 Hz, 1H), 3.69 (t, *J =* 10.4 Hz, 1H). ^13^C-NMR (CDCl_3_) δ 171.0, 169.0, 132.6, 132.3, 131.3, 130.7, 130.4, 126.7, 78.0, 52.8, 36.4. ESI-MS *m*/*z*: Calcd for C_11_H_10_ClNO_2_S: 256.01:258.01 = 3:1 [M + H]^+^; found: 256.27:258.19 = 3:1. ${\left[\alpha \right]}_{\text{D}}^{20}$ +32.4° (*c* = 0.1, MeOH).

*(S)-2-(2′-Bromophenyl)-4-methoxycarbonyl-4,5-dihydrothiazole* (**10e**). Yellow oil. ^1^H-NMR (CDCl_3_) δ 7.64–7.62 (m, 1H), 7.65–7.55 (m, 1H), 7.37–7.29 (m, 2H), 5.35–5.31 (m, 1 H), 3.86–3.84 (m, 3H), 3.81–3.69 (m, 2H). ^13^C-NMR (CDCl_3_) δ 175.5, 170.9, 170.2, 134.5, 133.6, 131.4, 130.6, 127.2, 121.3, 78.3, 52.8, 36.5. ESI-MS *m*/*z*: Calcd for C_11_H_10_BrNO_2_S: 299.96:301.96 = 1:1 [M + H]^+^; found: 299.88:301.76 = 1:1. ${\left[\alpha \right]}_{\text{D}}^{20}$ +22.0° (*c* = 0.1, MeOH).

*(R)-2-Phenyl-4-methoxycarbonyl-4,5-dihydrothiazole* (**1e′**). White powder. m.p. 60–62 °C. ^1^H-NMR (CDCl_3_) δ 7.87 (d, *J =* 7.3 Hz, 2H), 7.48 (t, *J =* 7.4 Hz, 1H), 7.41 (t, *J =* 7.6 Hz, 2H), 5.30 (t, *J =* 9.1 Hz, 1H), 3.84 (s, 3H), 3.73 (dd, *J =* 8.8, 11.0 Hz, 1H), 3.66–3.62 (m, 1H). ^13^C-NMR (CDCl_3_) δ 171.3, 170.9, 132.6, 131.6, 128.6 × 2, 128.5 × 2, 78.5, 52.7, 35.3. ESI-MS *m*/*z*: Calcd for C_11_H_11_NO_2_S: 222.05 [M + H]^+^; found: 222.24. ${\left[\alpha \right]}_{\text{D}}^{20}$ −129.0° (*c* = 0.1, MeOH).

*(R)-2-(4′-Hydroxyphenyl)-4-methoxycarbonyl-4,5-dihydrothiazole* (**2e′**). White powder. m.p. 166–168 °C. ^1^H-NMR (CDCl_3_) δ 7.70–7.69 (m, 2H), 6.78–6.76 (m, 2H), 5.28 (t, *J =* 9.0 Hz, 1H), 3.79 (s, 3H), 3.72–3.62 (m, 2H). ^13^C-NMR (CDCl_3_) δ 172.1, 171.5, 159.7, 130.6 × 2, 124.4, 115.5 × 2, 77.6, 52.8, 35.3. ESI-MS *m*/*z*: Calcd for C_11_H_11_NO_3_S: 238.05 [M + H]^+^; found: 238.05. ${\left[\alpha \right]}_{\text{D}}^{20}$ −109.6° (*c* = 0.1, MeOH).

*(R)-2-(4′-Fluorophenyl)-4-methoxycarbonyl-4,5-dihydrothiazole* (**3e′**). Yellow oil. ^1^H-NMR (CDCl_3_) δ 7.89–7.86 (m, 2H), 7.10 (t, *J =* 8.5 Hz, 2H), 5.28 (t, *J =* 9.1 Hz, 1H), 3.84 (s, 3H), 3.75–3.63 (m, 2H) ^13^C-NMR (CDCl_3_) δ 171.2, 169.6, 164.8 (d, *J =* 252 Hz), 130.8, 130.7, 128.9, 115.7, 115.5, 78.4, 52.8, 35.6. ESI-MS *m*/*z*: Calcd for C_11_H_10_FNO_2_S: 240.04 [M + H]^+^; found: 240.18. ${\left[\alpha \right]}_{\text{D}}^{20}$ −57.8° (*c* = 0.1, MeOH).

*(R)-2-(4′-Bromophenyl)-4-methoxycarbonyl-4,5-dihydrothiazole* (**4e′**). White solid. m.p. 74–76 °C. ^1^H-NMR (CDCl_3_) δ 7.75–7.73 (m, *J =* 8.2 Hz, 2H), 7.57–7.55 (m, *J =* 8.2 Hz, 2H), 5.28 (t, *J =* 9.1 Hz, 1H), 3.85 (s, 3H), 3.76–3.72 (m, 1H), 3.68–3.64 (m, 1H). ^13^C-NMR (CDCl_3_) δ 171.2, 170.0, 131.8 × 2, 131.6, 130.1 × 2, 126.4, 78.5, 52.9, 35.7. ESI-MS *m*/*z*: Calcd for C_11_H_10_BrNO_2_S: 299.96:301.96 = 1:1 [M + H]^+^; found: 299.79:301.86 = 1:1. ${\left[\alpha \right]}_{\text{D}}^{20}$ −7.6° (*c* = 0.1, MeOH).

*(R)-2-(3′-Hydroxyphenyl)-4-methoxycarbonyl-4,5-dihydrothiazole* (**5e′**). Colourless oil. ^1^H-NMR (CDCl_3_) δ 7.38 (s, 1H), 7.32 (d, *J* = 7.6 Hz, 1H), 7.23 (t, *J* = 7.9 Hz, 1H), 6.98–6.96 (m, 1H), 5.29 (t, *J =* 9.1 Hz, 1H), 3.81 (s, 3H), 3.72–3.62 (m, 2H). ^13^C-NMR (CDCl_3_) δ 172.2, 171.3, 156.2, 133.5, 129.8, 121.0, 119.4, 114.8, 77.9, 52.9, 35.2. ESI-MS *m*/*z*: Calcd for C_11_H_11_NO_3_S: 238.05 [M + H]^+^; found: 238.24. ${\left[\alpha \right]}_{\text{D}}^{20}$ −50.2° (*c* = 0.1, MeOH).

*(R)-2-(3′-Methylphenyl)-4-methoxycarbonyl-4,5-dihydrothiazole* (**6e′**). Colourless oil. ^1^H-NMR (CDCl_3_) δ 7.72 (s, 1H), 7.65–7.64 (m, 1H), 7.30 (d, *J =* 4.7 Hz, 2H), 5.29 (t, *J =* 9.1 Hz, 1H), 3.84 (s, 3H), 3.74–3.61 (m, 2H), 2.39 (s, 3H). ^13^C-NMR (CDCl_3_) δ 171.3, 171.2, 138.3, 132.5, 132.5, 128.9, 128.4, 125.9, 78.4, 52.7, 35.3, 21.2. ESI-MS *m*/*z*: Calcd for C_12_H_13_NO_2_S: 236.07 [M + H]^+^; found: 236.16. ${\left[\alpha \right]}_{\text{D}}^{20}$ −71.7° (*c* = 0.1, MeOH).

*(R)-2-(2′-Hydroxyphenyl)-4-methoxycarbonyl-4,5-dihydrothiazole* (**7e′**). Colorless oil. ${\left[\alpha \right]}_{\text{D}}^{20}$ −13.9° (*c* = 0.1, MeOH) [3].

*(R)-2-(2′-Fluorophenyl)-4-methoxycarbonyl-4,5-dihydrothiazole* (**8e′**). White solid. m.p. 70–72 °C. ^1^H-NMR (CDCl_3_) δ 7.95–7.92 (m, 1H), 7.47–7.42 (m, 1H), 7.20–7.11 (m, 2H), 5.25 (t, *J* = 9.3 Hz, 1H), 3.84 (s, 3H), 3.74–3.62 (m, 2H). ^13^C-NMR (CDCl_3_) δ 171.2, 165.9 (d, *J =* 5.04 Hz), 160.6 (d, *J* = 255.78 Hz), 133.0 (d, *J =* 8.82 Hz), 130.7 (d, *J =* 2.52 Hz), 124.1 (d, *J* = 2.52 Hz), 120.7 (d, *J =* 10.08 Hz), 116.3 (d, *J =* 22.68 Hz), 77.2, 52.8, 35.3 (d, *J* = 3.78 Hz). ESI-MS *m*/*z*: Calcd for C_11_H_10_FNO_2_S: 240.04 [M + H]^+^; found: 240.25. ${\left[\alpha \right]}_{\text{D}}^{20}$ −14.4° (*c* = 0.1, MeOH).

*(R)-2-(2′-Chlorophenyl)-4-methoxycarbonyl-4,5-dihydrothiazole* (**9e′**). Yellow oil. ^1^H-NMR (CDCl_3_) δ 7.65 (dd, *J =* 1.6, 7.6 Hz, 1H), 7.44–7.42 (m, 1H), 7.37–7.29 (m, 2H), 5.30 (t, *J =* 9.3 Hz, 1H), 3.85 (s, 3 H), 3.79 (dd, *J =* 9.1, 11.0 Hz, 1H), 3.69 (dd, *J =* 9.6, 11.2 Hz, 1H). ^13^C-NMR (CDCl_3_) δ 171.0, 169.0, 132.6, 132.3, 131.3, 130.7, 130.4, 126.7, 78.0, 52.8, 36.3. ESI-MS *m*/*z*: Calcd for C_11_H_10_ClNO_2_S: 256.01:258.01 = 3:1 [M + H]^+^; found: 256.12:258.37 = 3:1. ${\left[\alpha \right]}_{\text{D}}^{20}$ −32.4° (*c* = 0.1, MeOH).

*(R)-2-(2′-Bromophenyl)-4-methoxycarbonyl-4,5-dihydrothiazole* (**10e′**). Yellow oil. ^1^H-NMR (CDCl_3_) δ 7.64–7.54 (m, 2H), 7.37–7.24 (m, 2H), 5.35–5.29 (m, 1H), 3.85 (s, 3H), 3.83–3.67 (m, 2H). ^13^C-NMR (CDCl_3_) δ 170.9, 170.1, 134.5, 133.6, 131.4, 130.6, 127.2, 121.3, 78.2, 52.8, 36.5. ESI-MS *m*/*z*: Calcd for C_11_H_10_BrNO_2_S: 299.96:301.96 = 1:1 [M + H]^+^; found: 299.92:301.76 = 1:1. ${\left[\alpha \right]}_{\text{D}}^{20}$ −22.0° (*c* = 0.1, MeOH).

#### 3.2.2. Synthesis of **1h**–**10h** and **1h′**–**10h′**

NaBH_4_ (52.8 mg, 1.1 mmol) was added slowly in parts to a stirred solution of **1e**–**10e** (1 mmol) in EtOH (10 mL). After 1 h, the reaction was complete (checked by TLC). Ethyl acetate was added, and then the mixture was washed with saturated aq. NaHCO_3_ (2 × 5 mL), H_2_O (2 × 5 mL), saturated aq. NaCl (2 × 5 mL), and dried with anhydrous Na_2_SO_4_. The mixture was concentrated under vacuum, and the crude product was chromatographed on silica gel to obtain the products **1e**–**10e** and **1e′**–**10e′**. The yields of **1h**–**10h** and **1h′**–**10h′** were from 76% to 89%.

*(S)-2-Phenyl-4-hydroxymethyly-4,5-dihydrothiazole* (**1h**). Yellow solid. m.p. 76–78 °C. ^1^H-NMR (CDCl_3_) δ 7.81 (d, *J* = 7.6 Hz, 2H), 7.48–7.39 (m, 3H), 4.83–4.77 (m, 1H), 4.02 (dd, *J =* 4.6, 11.2 Hz, 1H), 3.80 (dd, *J =* 5.7, 11.3 Hz, 1H), 3.46–3.42 (m, 1H), 3.33–3.29 (m, 1H). ^13^C-NMR (CDCl_3_) δ 169.7, 132.9, 131.4, 128.5 × 2, 128.4 × 2, 79.3, 64.5, 34.4. ESI-MS *m*/*z*: Calcd for C_10_H_11_NOS: 194.06 [M + H]^+^; found: 194.22. ${\left[\alpha \right]}_{\text{D}}^{20}$ +30.7° (*c* = 0.1, MeOH).

*(S)-2-(4′-Hydroxyphenyl)-4-hydroxymethyl-4,5-dihydrothiazole* (**2h**). White solid. m.p. 196–198 °C. ^1^H-NMR (DMSO-*d*_6_) δ 10.06 (s, 1H), 7.59 (d, *J* = 8.5 Hz, 2H), 6.81 (d, *J =* 8.5 Hz, 2H), 4.91 (t, *J =* 5.4 Hz, 1H), 4.66–4.60 (m, 1H), 3.68–3.64 (m, 1H), 3.50–3.40 (m, 2H), 3.30–3.27 (m, 1H). ^13^C-NMR (DMSO-*d*_6_) δ 165.3, 160.2, 129.9 × 2, 124.0, 115.3 × 2, 79.3, 62.3, 34.5. ESI-MS *m*/*z*: Calcd for C_10_H_11_NO_2_S: 210.05 [M + H]^+^; found: 210.23. ${\left[\alpha \right]}_{\text{D}}^{20}$ +124.0° (*c* = 0.1, MeOH).

*(S)-2-(4′-Fluorophenyl)-4-hydroxymethyly-4,5-dihydrothiazole* (**3h**). White solid. m.p. 60–62 °C. ^1^H-NMR (CDCl_3_) δ 7.79–7.76 (m, 2H), 7.07 (t, *J* = 10 Hz, 2H), 4.79–4.74 (m, 1H), 4.03 (dd, *J* = 4.4, 11.3 Hz, 1H), 3.78 (dd, *J* = 5.5, 11.2 Hz, 1H), 3.43 (dd, *J* = 9.0, 10.9 Hz, 1H), 3.35–3.31 (m, 1H). ^13^C-NMR (CDCl_3_) δ 168.4, 164.6 (d, *J =* 252 Hz) 130.5, 130.4, 129.1, 115.6, 115.4, 79.3, 64.3, 34.6. ESI-MS *m*/*z*: Calcd for C_10_H_10_FNOS: 212.05 [M + H]^+^; found: 212.31. ${\left[\alpha \right]}_{\text{D}}^{20}$ +10.9° (*c* = 0.1, MeOH).

*(S)-2-(4′-Bromophenyl)-4-hydroxymethyl-4,5-dihydrothiazole* (**4h**). White solid. m.p. 96–98 °C. ^1^H-NMR (CDCl_3_) δ 7.67–7.65 (m, 2H), 7.54–7.52 (m, 2H), 4.80–4.75 (m, 1H), 4.03 (dd, *J* = 4.7, 11.3 Hz, 1H), 3.79 (dd, *J* = 5.7, 11.3 Hz, 1H), 3.46 (dd, *J* = 8.8, 10.7 Hz, 1H), 3.33 (dd, *J* = 9.3, 10.9 Hz, 1H). ^13^C-NMR (CDCl_3_) δ 168.7, 131.8, 131.7 × 2, 129.8 × 2, 126.0, 79.4, 64.4, 34.6. ESI-MS *m*/*z*: Calcd for C_10_H_10_BrNOS: 271.97:273.97 = 1:1 [M + H]^+^; found: 272.16:274.04 = 1:1. ${\left[\alpha \right]}_{\text{D}}^{20}$ +26.6° (*c* = 0.1, MeOH).

*(S)-2-(3′-Hydroxyphenyl)-4-hydroxymethyl-4,5-dihydrothiazole* (**5h**). White solid. m.p. 118–120 °C. ^1^H-NMR (CD_3_OD) δ 7.27–7.23 (m, 3H), 6.98–6.96(m, 1H), 4.78–4.73 (m, 1H), 4.04 (s, 2H), 3.85–3.78 (m, 2H). ^13^C-NMR (CD_3_OD) δ 171.1, 156.8, 133.6, 129.5, 119.7, 118.7, 114.2, 78.5, 63.1, 34.2. ESI-MS *m*/*z*: Calcd for C_10_H_11_NO_2_S: 210.05 [M + H]^+^; found: 210.27. ${\left[\alpha \right]}_{\text{D}}^{20}$ +178.2° (*c* = 0.1, MeOH).

*(S)-2-(3′-Methylphenyl)-4-hydroxymethyl-4,5-dihydrothiazole* (**6h**). Yellow oil. ^1^H-NMR (CDCl_3_) δ 7.62–7.57 (m, 2H), 7.28–7.27 (m, 2H), 4.81–4.75 (m, 1H), 4.02 (dd, *J* = 4.7, 11.0 Hz, 1H), 3.79 (dd, *J* = 5.5, 11.2 Hz, 1H), 3.42 (dd, *J* = 8.8, 10.7 Hz, 1H), 3.31 (dd, *J* = 9.5, 10.7 Hz, 1H), 2.38 (s, 3H). ^13^C-NMR (CDCl_3_) δ 169.9, 138.2, 132.8, 132.1, 128.8, 128.3, 125.6, 79.3, 64.4, 34.3, 21.2. ESI-MS *m*/*z*: Calcd for C_11_H_13_NOS: 208.07 [M + H]^+^; found: 208.32. ${\left[\alpha \right]}_{\text{D}}^{20}$ +7.6° (*c* = 0.1, MeOH).

*(S)-2-(2′-Hydroxyphenyl)-4-hydroxymethyl-4,5-dihydrothiazole* (**7h**). White solid. m.p. 43–45 °C. ${\left[\alpha \right]}_{\text{D}}^{20}$ +16.5° (*c* = 0.1, MeOH) [3].

*(S)-2-(2′-Fluorophenyl)-4-hydroxymethyl-4,5-dihydrothiazole* (**8h**). White solid. m.p. 78–80 °C. ^1^H-NMR (CDCl_3_) δ 7.86–7.82 (m, 1H), 7.46–7.41 (m, 1H), 7.20–7.11 (m, 2H), 4.81–4.75 (m, 1H), 4.00 (dd, *J* = 4.9, 11.2 Hz, 1H), 3.80 (dd, *J* = 5.7, 11.3 Hz, 1H), 3.44 (dd, *J* = 9.1, 10.7 Hz, 1H), 3.30 (dd, *J* = 9.1, 10.7 Hz, 1H). ^13^C-NMR (CDCl_3_) δ 164.6 (d, *J =* 5.04 Hz), 160.3 (d, *J =* 255.78 Hz), 132.6 (d, *J =* 8.82 Hz), 130.5 (d, *J =* 1.26 Hz), 124.1 (d, *J =* 3.78 Hz), 121.1 (d, *J =* 10.1 Hz), 116.4 (d, *J =* 25.2 Hz), 78.4, 64.4, 34.4. ESI-MS *m*/*z*: Calcd for C_10_H_10_FNOS: 212.05 [M + H]^+^; found: 212.41. ${\left[\alpha \right]}_{\text{D}}^{20}$ +25.9° (*c* = 0.1, MeOH).

*(S)-2-(2′-Chlorophenyl)-4-hydroxymethyl-4,5-dihydrothiazole* (**9h**). Yellow oil. ^1^H-NMR (CDCl_3_) δ 7.57 (dd, *J* = 1.6, 7.6 Hz, 1H), 7.44–7.42 (m, 1H), 7.36–7.29 (m, 2H), 4.85–4.80 (m, 1H), 3.95 (dd, *J* = 4.9, 11.2 Hz, 1H), 3.78 (dd, *J* = 5.5, 11.2 Hz, 1H), 3.48 (dd, *J* = 9.1, 10.7 Hz, 1H), 3.36 (dd, *J* = 8.7, 10.9 Hz, 1H). ^13^C-NMR (CDCl_3_) δ 167.5, 132.7, 132.1, 131.1, 130.4, 130.3, 126.7, 79.2, 64.2, 35.5. ESI-MS *m*/*z*: Calcd for C_10_H_10_ClNOS: 228.02:230.02 = 3:1 [M + H]^+^; found: 228.21:230.37 = 3:1. ${\left[\alpha \right]}_{\text{D}}^{20}$ +19.4° (*c* = 0.1, MeOH).

*(S)-2-(2′-Bromophenyl)-4-hydroxymethyl-4,5-dihydrothiazole* (**10h**). Yellow solid. m.p. 54–56 °C. ^1^H-NMR (CDCl_3_) δ 7.64 (d, *J =* 7.9 Hz, 1H), 7.51 (d, *J =* 7.6 Hz, 1H), 7.37–7.29 (m, 2H), 4.91–4.86 (m, 1H), 4.04–4.01 (m, 1H), 3.81 (dd, *J =* 5.5, 11.2 Hz, 1H), 3.55–3.51 (m, 1H), 3.42 (dd, *J =* 8.8, 10.7 Hz, 1H). ^13^C-NMR (CDCl_3_) δ 168.7, 134.8, 133.6, 131.2, 130.2, 127.3, 121.0, 79.5, 64.5, 35.7. ESI-MS *m*/*z*: Calcd for C_10_H_10_BrNOS 271.97:273.97 = 1:1 [M + H]^+^; found: 271.86:273.91 = 1:1. ${\left[\alpha \right]}_{\text{D}}^{20}$ +109.1° (*c* = 0.1, MeOH).

*(R)-2-Phenyl-4-hydroxymethyl-4,5-dihydrothiazole* (**1h′**). Yellow solid. m.p. 76–78 °C. ^1^H-NMR (CDCl_3_) δ 7.79 (d, *J =* 7.9 Hz, 2H), 7.47–7.37 (m, 4H), 4.80–4.75 (m, 1H), 4.01 (dd, *J =* 4.7, 11.0 Hz, 1H), 3.79 (dd, *J =* 5.5, 11.2 Hz, 1H), 3.44–3.40 (m, 1H), 3.33–3.29 (m, 1H), 2.78 (br. s, 1H). ^13^C-NMR (CDCl_3_) δ 169.8, 132.8, 131.4, 128.4 × 2, 128.3 × 2, 79.3, 64.3, 34.3. ESI-MS *m*/*z*: Calcd for C_10_H_11_NO_2_S: 194.06 [M + H]^+^; found: 194.23. ${\left[\alpha \right]}_{\text{D}}^{20}$ −30.6° (*c* = 0.1, MeOH).

*(R)-2-(4′-Hydroxyphenyl)-4-hydroxymethyl-4,5-dihydrothiazole* (**2h′**). White solid. m.p. 196–198 °C. ^1^H-NMR (DMSO-*d*_6_) δ 10.12 (s, 1H), 7.75–7.60 (m, 2H), 6.95–6.80 (m, 2H), 4.97 (t, *J =* 5.7 Hz, 1H), 4.72–4.66 (m, 1H), 3.75–3.70 (m, 1H), 3.56–3.51 (m, 1H), 3.48 (dd, *J =* 8.7, 10.9 Hz, 1H), 3.35 (dd, *J =* 7.4, 10.9 Hz, 1H). ^13^C-NMR (DMSO-*d*_6_) δ 165.3, 160.2, 129.8 × 2, 124.0, 115.3 × 2, 79.3, 62.3, 34.5. ESI-MS *m*/*z*: Calcd for C_11_H_11_NO_2_S: 210.05 [M + H]^+^; found: 210.16. ${\left[\alpha \right]}_{\text{D}}^{20}$ −123.9° (*c* = 0.1, MeOH).

*(R)-2-(4′-Fluorophenyl)-4-hydroxymethyl-4,5-dihydrothiazole* (**3h′**). White solid. m.p. 60–62 °C. ^1^H-NMR (CDCl_3_) δ 7.81 (dd, *J =* 5.4, 8.8 Hz, 2H), 7.08 (t, *J =* 8.5 Hz, 2H), 4.81–4.75 (m, 1H), 4.02 (dd, *J =* 4.6, 11.2 Hz, 1H), 3.79 (dd, *J =* 5.7, 11.0 Hz, 1H), 3.45 (dd, *J =* 8.8, 10.7 Hz, 1H), 3.34–3.30 (m, 1H). ^13^C-NMR (CDCl_3_) δ 168.3, 164.6 (d, *J =* 252 Hz), 130.5, 130.5, 129.2, 115.6, 115.5, 79.3, 64.5, 34.7. ESI-MS *m*/*z*: Calcd for C_10_H_10_FNOS: 212.05 [M + H]^+^; found: 212.11. ${\left[\alpha \right]}_{\text{D}}^{20}$ −10.9° (*c* = 0.1, MeOH).

*(R)-2-(4′-Bromophenyl)-4-hydroxymethyl-4,5-dihydrothiazole* (**4h′**). White solid. m.p. 96–98 °C. ^1^H-NMR (CDCl_3_) δ 7.68–7.66 (m, 2H), 7.55–7.53 (m, 2H), 4.81–4.76 (m, 1H), 4.03 (dd, *J =* 4.7, 11.0 Hz, 1H), 3.79 (dd, *J =* 5.7, 11.3 Hz, 1H), 3.46 (dd, *J =* 8.8, 10.7 Hz, 1H), 3.33 (dd, *J =* 9.3, 10.9 Hz, 1H). ^13^C-NMR (CDCl_3_) δ 168.5, 131.8, 131.7 × 2, 129.8 × 2, 126.0, 79.4, 64.5, 34.6. ESI-MS *m*/*z*: Calcd for C_10_H_10_BrNOS: 271.97:273.97 = 1:1 [M + H]^+^; found: 271.82:273.77 = 1:1. ${\left[\alpha \right]}_{\text{D}}^{20}$ −26.6° (*c* = 0.1, MeOH).

*(R)-2-(3′-Hydroxyphenyl)-4-hydroxymethyl-4,5-dihydrothiazole* (**5h′**). White solid. m.p. 118–120 °C. ^1^H-NMR (CDCl_3_) δ 7.35 (br. s, 1H), 7.27–7.24 (m, 2H), 6.97–6.96 (m, 1H), 4.75–4.74 (m, 1H), 3.82–3.80 (m, 2H), 3.62–3.61 (m, 2H). ^13^C-NMR (CDCl_3_) δ 171.3, 156.8, 133.6, 129.6, 120.0, 118.9, 114.0, 78.5, 63.3, 34.2. ESI-MS *m*/*z*: Calcd for C_10_H_11_NO_2_S: 210.05 [M + H]^+^; found: 210.23. ${\left[\alpha \right]}_{\text{D}}^{20}$ −178.2° (*c* = 0.1, MeOH).

*(R)-2-(3′-Methylphenyl)-4-hydroxymethyl-4,5-dihydrothiazole* (**6h′**). Yellow oil. ^1^H-NMR (CDCl_3_) δ 7.61–7.56 (m, 2H), 7.29–7.26 (m, 2H), 4.79–4.74 (m, 1H), 4.01 (dd, *J =* 4.7, 11.3 Hz, 1H), 3.78 (dd, *J =* 5.5, 11.2 Hz, 1H), 3.41 (dd, *J =* 8.8, 10.7 Hz, 1H), 3.30 (dd, *J =* 9.1, 10.7 Hz, 1H), 2.37 (s, 3H). ^13^C-NMR (CDCl_3_) δ 169.9, 138.2, 132.7, 132.1, 128.8, 128.3, 125.6, 79.2, 64.3, 34.2, 21.2. ESI-MS *m*/*z*: Calcd for C_11_H_13_NOS: 208.07 [M + H]^+^; found: 208.01. ${\left[\alpha \right]}_{\text{D}}^{20}$ −7.6° (*c* = 0.1, MeOH).

*(R)-2-(2′-Hydroxyphenyl)-4-hydroxymethyl-4,5-dihydrothiazole* (**7h′**). White solid. m.p. 44–46 °C. ${\left[\alpha \right]}_{\text{D}}^{20}$ −16.1° (*c* = 0.1, MeOH) [3].

*(R)-2-(2′-Fluorophenyl)-4-hydroxymethyl-4,5-dihydrothiazole* (**8h′**). White solid. m.p. 78–80 °C. ^1^H-NMR (CDCl_3_) δ 7.94–7.75 (m, 1H), 7.57–7.34 (m, 1H), 7.24–7.02 (m, 2H), 4.86–4.71 (m, 1H), 4.00 (dd, *J =* 4.7, 11.3 Hz, 1H), 3.80 (dd, *J =* 5.7, 11.0 Hz, 1H), 3.44 (dd, *J =* 9.0, 10.9 Hz, 1H), 3.31 (dd, *J =* 9.5, 10.7 Hz, 1H), 2.62 (br. s, 1H). ^13^C-NMR (CDCl_3_) δ 164.6 (d, *J =* 5.04 Hz), 160.4, (d, *J =* 255.78 Hz), 132.6 (d, *J =* 8.82 Hz), 130.5 (d, *J =* 2.52 Hz), 124.1 (d, *J =* 3.78 Hz), 121.1 (d, *J =* 11.34 Hz), 116.4 (d, *J =* 22.68 Hz), 78.4, 64.4, 34.4 (d, *J =* 2.52 Hz). ESI-MS *m*/*z*: Calcd for C_10_H_10_FNOS: 212.05 [M + H]^+^; found: 212.29. ${\left[\alpha \right]}_{\text{D}}^{20}$ −25.9° (*c* = 0.1, MeOH).

*(R)-2-(2′-Chlorophenyl)-4-hydroxymethyl-4,5-dihydrothiazole* (**9h′**). Yellow oil. ^1^H-NMR (CDCl_3_) δ 7.58 (d, *J =* 7.6 Hz, 1H), 7.44 (d, *J =* 7.6 Hz, 1H), 7.36–7.28 (m, 2H), 4.86–4.80 (m, 1H), 3.96 (dd, *J =* 4.9, 11.2 Hz, 1H), 3.81–3.77 (m, 1H), 3.53–3.47 (m, 1H), 3.41–3.35 (m, 1H). ^13^C-NMR (CDCl_3_) δ 167.5, 132.7, 132.1, 131.1, 130.4, 130.4, 126.7, 79.2, 64.2, 35.5. ESI-MS *m*/*z*: Calcd for C_10_H_10_ClNOS: 228.02:230.02 = 3:1 [M + H]^+^; found: 228.16:230.27 = 3:1. ${\left[\alpha \right]}_{\text{D}}^{20}$ −19.4° (*c* = 0.1, MeOH).

*(R)-2-(2′-Bromophenyl)-4-hydroxymethyl-4,5-dihydrothiazole* (**10h′**). Yellow solid. m.p. 54–56 °C. ^1^H-NMR (CDCl_3_) δ 7.69–7.63 (m, 1H), 7.55–7.50 (m, 1H), 7.36–7.28 (m, 1H), 4.90–4.85 (m, 1H), 4.03–3.95 (m, 1H), 3.83–3.76 (m, 1H), 3.55–3.51 (m, 1H), 3.46–3.39 (m, 1H). ^13^C-NMR (CDCl_3_) δ 168.7, 133.6, 131.7, 131.2, 130.2, 129.8, 127.3, 79.4, 64.4, 35.6. ESI-MS *m*/*z*: Calcd for C_10_H_10_BrNOS: 271.97:273.97 = 1:1 [M + H]^+^; found: 271.82:273.79 = 1:1. ${\left[\alpha \right]}_{\text{D}}^{20}$ −109.1° (*c* = 0.1, MeOH).

#### 3.2.3. Synthesis of **1a**–**10a** and **1a′**–**10a′**

10% sodium hydroxide solution was added slowly in parts to a stirred solution of **1e**–**10e** and **1e′**–**10e′** (1mmol) in EtOH (10 mL). After 2 h, the reaction was complete (checked by TLC). Purification of the crude reaction mixture by column chromatography on silica gel (PE/ EA) afforded the desired products and the yields were from 81% to 90%.

*(S)-2-Phenyl-4-carboxy-4,5-dihydrothiazole* (**1a**). Yellow powder. m.p. 116–118 °C. ^1^H-NMR (CDCl_3_) δ 7.83 (d, *J =* 7.6 Hz, 2H), 7.48–7.45 (m, 1H), 7.40–7.38 (m, 2H), 5.26 (t, *J =* 9.1 Hz, 1H), 3.72–3.64 (m, 2H). ^13^C-NMR (CDCl_3_) δ 172.6, 171.8, 132.4, 131.7, 128.9 × 2, 128.1 × 2, 78.1, 35.1. ESI-MS *m*/*z*: Calcd for C_10_H_9_NO_2_S: 208.04 [M + H]^+^; found: 208.25. ${\left[\alpha \right]}_{\text{D}}^{20}$ +10.5° (*c* = 0.1, MeOH).

*(S)-2-(4′-Hydroxyphenyl)-4-carboxy-4,5-dihydrothiazole* (**2a**). White solid. m.p. 150–152 °C. ${\left[\alpha \right]}_{\text{D}}^{20}$ +12.5° (*c* = 0.1, MeOH) [13].

*(S)-2-(4′-Fluorophenyl)-4-carboxy-4,5-dihydrothiazole* (**3a**). White solid. m.p. 120–122 °C. ^1^H-NMR (CDCl_3_) δ 9.14 (br. s, 1 H), 7.87 (dd, *J =* 5.4, 8.5 Hz, 2H), 7.12 (t, *J =* 8.5 Hz, 2H), 5.38 (t, *J =* 9.1 Hz, 1H), 3.78–3.70 (m, 2H). ^13^C-NMR (CDCl_3_) δ 173.6, 172.0, 165.1 (d, *J =* 252 Hz), 130.9, 130.9, 128.3, 115.9, 115.7, 77.6, 35.3. ESI-MS *m*/*z*: Calcd for C_10_H_8_FNO_2_S: 226.03 [M + H]^+^; found: 226.08. ${\left[\alpha \right]}_{\text{D}}^{20}$ +25.3° (*c* = 0.1, MeOH).

*(S)-2-(4′-Bromophenyl)-4-carboxy-4,5-dihydrothiazole* (**4a**). White power. m.p. 194–196 °C. ^1^H-NMR (CDCl_3_) δ 7.74–7.72 (m, 2H), 7.59–7.58 (m, 2H), 5.35 (t, *J =* 9.6 Hz, 1H), 3.76 (d, *J =* 9.8 Hz, 2H). ^13^C-NMR (CDCl_3_) δ 172.5, 171.9, 132.0 × 2, 130.9, 130.0 × 2, 127.0, 78.0, 35.2. ESI-MS *m*/*z*: Calcd for C_10_H_8_BrNO_2_S: 285.95:287.95 = 1:1 [M + H]^+^; found: 285.91:287.75 = 1:1. ${\left[\alpha \right]}_{\text{D}}^{20}$ +14.6° (*c* = 0.1, MeOH).

*(S)-2-(3′-Hydroxyphenyl)-4-carboxy-4,5-dihydrothiazole* (**5a**). Yellow oil. ${\left[\alpha \right]}_{\text{D}}^{20}$ +13.3° (*c* = 0.1, MeOH) [14].

*(S)-2-(3′-Methylphenyl)-4-carboxy-4,5-dihydrothiazole* (**6a**). Yellow power. m.p. 118–120 °C. ^1^H-NMR (CDCl_3_) δ 7.66 (br. s, 1H), 7.64–7.49 (m, 1H), 7.48–7.27 (m, 2H), 5.25 (t, *J =* 9.1 Hz, 1H), 3.70–3.61 (m, 2H), 2.36 (s, 3H). ^13^C-NMR (CDCl_3_) δ 172.6, 172.0, 138.2, 132.5, 132.2, 128.8, 128.3, 125.6, 78.0, 35.0, 21.0. ESI-MS *m*/*z*: Calcd for C_11_H_11_NO_2_S: 222.05 [M + H]^+^; found: 222.33. ${\left[\alpha \right]}_{\text{D}}^{20}$ +30.2° (*c* = 0.1, MeOH).

*(S)-2-(2′-Hydroxyphenyl)-4-carboxy-4,5-dihydrothiazole* (**7a**). Yellow solid. m.p. 128–130 °C. ${\left[\alpha \right]}_{\text{D}}^{20}$ +159.9° (*c* = 0.1, MeOH) [15].

*(S)-2-(2′-Fluorophenyl)-4-carboxy-4,5-dihydrothiazole* (**8a**). Yellow oil. ^1^H-NMR (DMSO-*d*_6_) δ 7.90–7.87 (m, 1H), 7.69–7.57 (m, 2H), 7.31–7.27 (m, 1H), 5.26 (t, *J =* 9.0 Hz, 1H), 3.73–3.68 (m, 1H), 3.63–3.59 (m, 1H). ^13^C-NMR (DMSO-*d*_6_) δ 171.7, 171.4, 163.1(d, *J =* 3.78 Hz), 133.6(d, *J =* 7.56 Hz), 130.3(d, *J =* 1.26 Hz), 124.9 (d, *J =* 2.52 Hz), 120.2(d, *J =* 7.56 Hz), 116.6(d, *J =* 8.82 Hz), 77.2, 35.0(d, *J =* 5.04 Hz). ESI-MS *m*/*z*: Calcd for C_11_H_11_NO_2_S: 226.03 [M + H]^+^; found: 226.03. ${\left[\alpha \right]}_{\text{D}}^{20}$ +96.8° (*c* = 0.1, MeOH).

*(S)-2-(2′-Chlorophenyl)-4-carboxy-4,5-dihydrothiazole* (**9a**). Yellow solid. m.p. 120–122 °C. ^1^H-NMR (CDCl_3_) δ 7.63 (d, *J =* 7.6 Hz, 1H), 7.46–7.43 (m, 1H), 7.39 (d, *J =* 7.9 Hz, 1H), 7.32–7.28 (m, 1H), 5.38 (t, *J =* 9.5 Hz, 1H), 3.92–3.68 (m, 2H). ^13^C-NMR (CDCl_3_) δ 173.1, 171.4, 132.5, 131.8, 131.5, 130.8, 130.6, 126.9, 77.5, 36.0. ESI-MS *m*/*z*: Calcd for C_10_H_8_ClNO_2_S: 242.00:244.00 = 3:1 [M + H]^+^; found: 242.36:244.48 = 3:1. ${\left[\alpha \right]}_{\text{D}}^{20}$ +62.6° (*c* = 0.1, MeOH).

*(S)-2-(2′-Bromophenyl)-4-carboxy-4,5-dihydrothiazole* (**10a**). Yellow oil. ^1^H-NMR (CDCl_3_) δ 7.65 (d, *J =* 8.2 Hz, 1H), 7.59–7.54 (m, 1H), 7.38–7.22 (m, 2H), 5.41 (t, *J =* 9.5 Hz, 1H), 3.87–3.76 (m, 2H). ^13^C-NMR (CDCl_3_) δ 172.6, 167.5, 133.9, 131.9, 130.6, 129.7, 127.4, 121.2, 77.8, 36.1. ESI-MS *m*/*z*: Calcd for C_10_H_8_BrNO_2_S: 285.95:287.95 = 1:1 [M + H]^+^; found: 285.87:287.92 = 1:1. ${\left[\alpha \right]}_{\text{D}}^{20}$ +74.5° (*c* = 0.1, MeOH).

*(R)-2-Phenyl-4-carboxy-4,5-dihydrothiazole* (**1a′**). Yellow powder. m.p. 116–118 °C. ^1^H-NMR (CDCl_3_) δ 7.83 (d, *J =* 7.6 Hz, 2H), 7.48–7.45 (m, 1H), 7.40–7.38 (m, 2H), 5.26 (t, *J =* 9.1 Hz, 1H), 3.72–3.64 (m, 2H). ^13^C-NMR (CDCl_3_) δ 172.6, 171.8, 132.4, 131.7, 128.9 × 2, 128.1 × 2, 78.1, 35.1. ESI-MS *m*/*z*: Calcd for C_10_H_9_NO_2_S: 208.04 [M + H]^+^; found: 208.26. ${\left[\alpha \right]}_{\text{D}}^{20}$ −10.5° (*c* = 0.1, MeOH).

*(R)-2-(4′-Hydroxyphenyl)-4-carboxy-4,5-dihydrothiazole* (**2a′**). White solid. m.p. 150–152 °C. ${\left[\alpha \right]}_{\text{D}}^{20}$ −12.5° (*c* = 0.1, MeOH) [13].

*(R)-2-(4′-Fluorophenyl)-4-carboxy-4,5-dihydrothiazole* (**3a′**). White solid. m.p. 120–122 °C. ^1^H-NMR (CDCl_3_) δ 10.23 (br. s, 1H), 7.86 (dd, *J =* 5.4, 8.5 Hz, 2H), 7.13–7.09 (m, 2H), 5.41–5.37 (m, 1H), 3.78–3.69 (m, 2H). ^13^C-NMR (CDCl_3_) δ 173.7, 172.0, 165.1 (d, *J =* 252 Hz), 130.9, 130.9, 128.3, 115.9, 115.7, 77.5, 35.3. ESI-MS *m*/*z*: Calcd for C_10_H_8_FNO_2_S: 226.04 [M + H]^+^; found: 226.33. ${\left[\alpha \right]}_{\text{D}}^{20}$ −25.3° (*c* = 0.1, MeOH).

*(R)-2-(4′-Bromophenyl)-4-carboxy-4,5-dihydrothiazole* (**4a′**). White power. m.p. 194–196 °C. ^1^H-NMR (DMSO-*d*_6_) δ 7.85–7.66 (m, 4H), 5.31 (t, *J* = 8.8 Hz, 1H), 3.84–3.70 (m, 1H), 3.70–3.59 (m, 1H). ^13^C-NMR (DMSO-*d*_6_) δ 171.6, 167.3, 131.9 × 2, 131.4, 130.0 × 2, 125.4, 78.4, 35.2. ESI-MS *m*/*z*: Calcd for C_10_H_8_BrNO_2_S: 285.95:287.95 = 1:1 [M + H]^+^; found: 285.92:287.86 = 1:1. ${\left[\alpha \right]}_{\text{D}}^{20}$ −14.6° (*c* = 0.1, MeOH).

*(R)-2-(3′-Hydroxyphenyl)-4-carboxy-4,5-dihydrothiazole* (**5a′**). Yellow oil. ${\left[\alpha \right]}_{\text{D}}^{20}$ −13.3° (*c* = 0.1, MeOH) [14].

*(R)-2-(3′-Methylphenyl)-4-carboxy-4,5-dihydrothiazole* (**6a′**). Yellow power. m.p. 118–120 °C. ^1^H-NMR (CDCl_3_) δ 7.67 (br. s, 1H), 7.64–7.54 (m, 1H), 7.40–7.27 (m, 2H), 5.25 (t, *J =* 9.1 Hz, 1H), 3.77–3.56 (m, 2H), 2.36 (s, 3H). ^13^C-NMR (CDCl_3_) δ 172.6, 172.0, 138.2, 132.5, 132.3, 128.9, 128.3, 125.7, 78.0, 35.0, 21.0. ESI-MS *m*/*z*: Calcd for C_11_H_11_NO_2_S: 222.05 [M + H]^+^; found: 222.27. ${\left[\alpha \right]}_{\text{D}}^{20}$ −30.2° (*c* = 0.1, MeOH).

*(R)-2-(2′-Hydroxyphenyl)-4-carboxy-4,5-dihydrothiazole* (**7a′**). Yellow solid. m.p. 128–130 °C. ${\left[\alpha \right]}_{\text{D}}^{20}$ −160.0° (*c* = 0.1, MeOH) [15].

*(R)-2-(2′-Fluorophenyl)-4-carboxy-4,5-dihydrothiazole* (**8a′**). Yellow oil. ^1^H-NMR (DMSO-*d*_6_) δ 7.90–7.87 (m, 1H), 7.69–7.57 (m, 2H), 7.31–7.27 (m, 1H), 5.26 (t, *J =* 9.0 Hz, 1H), 3.73–3.68 (m, 1H), 3.63–3.59 (m, 1H). ^13^C-NMR (DMSO-*d*_6_) δ 171.7, 171.4, 163.1 (d, *J =* 3.78 Hz), 133.6 (d, *J =* 7.56 Hz), 130.3(d, *J =* 1.26 Hz), 124.9 (d, *J =* 2.52 Hz), 120.2 (d, *J =* 7.56 Hz), 116.6 (d, *J =* 8.82 Hz), 77.2, 35.0 (d, *J =* 5.04 Hz). ESI-MS *m*/*z*: Calcd for C_10_H_8_FNO_2_S: 226.03 [M + H]^+^; found: 226.18. ${\left[\alpha \right]}_{\text{D}}^{20}$ −96.8° (*c* = 0.1, MeOH).

*(R)-2-(2′-Chlorophenyl)-4-carboxy-4,5-dihydrothiazole* (**9a′**). Yellow solid. m.p. 120–122 °C. ^1^H-NMR (CDCl_3_) δ 7.63 (d, *J =* 7.6 Hz, 1H), 7.46–7.43 (m, 1H), 7.39 (d, *J =* 7.9 Hz, 1H), 7.32–7.28 (m, 1H), 5.38 (t, *J =* 9.5 Hz, 1H), 3.92–3.68 (m, 2H). ^13^C-NMR (CDCl_3_) δ 173.1, 171.4, 132.5, 131.8, 131.5, 130.8, 130.6, 126.9, 77.5, 36.0. ESI-MS *m*/*z*: Calcd for C_10_H_8_ClNO_2_S: 242.00:244.00 = 3:1 [M + H]^+^; found: 242.06:244.12 = 3:1. ${\left[\alpha \right]}_{\text{D}}^{20}$ −62.6° (*c* = 0.1, MeOH).

*(R)-2-(2′-bromophenyl)-4-carboxy-4,5-dihydrothiazole* (**10a′**). Yellow oil. ^1^H-NMR (CDCl_3_) δ 7.65 (d, *J =* 8.2 Hz, 1H), 7.59–7.54 (m, 1H), 7.38–7.22 (m, 2H), 5.41 (t, *J =* 9.5 Hz, 1H), 3.87–3.76 (m, 2H). ^13^C-NMR (CDCl_3_) δ 172.6, 167.5, 133.9, 131.9, 130.6, 129.7, 127.4, 121.2, 77.8, 36.1. ESI-MS *m*/*z*: Calcd for C_10_H_8_BrNO_2_S: 285.95:287.95 = 1:1 [M + H]^+^; found: 285.87:287.69 = 1:1. ${\left[\alpha \right]}_{\text{D}}^{20}$ −74.5° (*c* = 0.1, MeOH).

### 3.3. Biology

#### 3.3.1. Filter Paper Assay

The standard bacterial stains were provided by the Institute of Plant Disease, Northwest Agriculture and Forestry University. Ampicillin (Sigma, Shanghai, China) was preferred as the positive control. Mueller–Hinton (Hangzhou Microbial Reagent Co., Ltd., Hangzhou, China) agar was used as an assay medium. The medium at 45 °C was mixed with a suspension containing the bacterial pathogen at approximately 108 colony forming units (CFU)·mL^−1^. Petri dishes (9 cm in diameter) were then flooded with the mixture. The tested samples were dissolved in acetone at the concentration of 1000 ppm, the filter papers (6 mm in diameter) were impregnated with 10 μL/disc of each compound, then were completely dried and placed on the inoculated agar. The inoculated plates were incubated at 37 °C for 10–12 h. Antibacterial activity was evaluated by measuring the zone of inhibition against the test organism. Experiments were run in triplicate.

#### 3.3.2. Minimal Inhibitory Concentration (MIC)

Antibacterial activities were measured by the micro-broth dilution method in 96-well culture plates using the Mueller–Hinton broth, according to the National Committee for Clinical Laboratory Standards. The tested bacteria were incubated in the Mueller–Hinton broth for 12 h at 37 °C at 190 rpm, and the spore concentration was diluted to approximately 1 × 10^5^–1 × 10^6^ CFU/mL with Mueller–Hinton broth. After incubation for 24 h at 37 °C, the MICs were examined by observing the first few holes transparent [3].

#### 3.3.3. The Fatty Acid Exposure Experiment

The medium at 45 °C was mixed with a suspension containing the bacterial pathogen at approximately 108 CFU·mL^−1^ and fatty acid or fatty acid salt of certain concentration gradient. Petri dishes (9 cm in diameter) were then flooded with the mixture. Compound **7h** was dissolved in acetone at the concentration of 100 ppm, the filter papers (6 mm in diameter) were impregnated with 10 μL/disc of each compound. The inoculated plates were incubated at 37 °C for 10–12 h. The fatty acid added experiment was evaluated by measuring the zone of inhibition against the test organism. The experiments were run in triplicate.

#### 3.3.4. Scanning Electron Microscopy

The tested bacteria were incubated in the Mueller–Hinton broth for 12 h at 37 °C at 180 rpm and the spore concentration was diluted to approximately 1 × 10^5^–1 × 10^6^ CFU/mL with Mueller–Hinton broth. Compound **7h** was dissolved in acetone and added to the broth. After incubation for 2 h at 37 °C then centrifuged at 3500 rpm for 20 min. All the cells were washed twice with 0.1 M phosphate buffer saline (PBS, pH 7.4) and fixed with 2.5% glutaraldehyde (*v*/*v*) in 0.1 M PBS at 4 °C for 24 h. Next, the cells were dehydrated using 30%, 50%, 70%, 90%, and 100% ethanol, and then the ethanol was replaced by tertiary butyl alcohol. The cells were dried at “critical point” in liquid CO_2_, and the samples were gold-covered by cathodic spraying before examination [16,17].

## 4. Conclusions

Aryl nitriles and methyl cysteine reacted in the presence of sodium carbonate in dry methanol to afford methyl-2-aryl-4, 5-dihydrothiazole-4-carboxylates in high yield. *Ortho* substituents did not favor the reaction, especially some large groups, such as -Br and -Cl. Meanwhile electron-withdrawing groups were better to this reaction than electron-donating ones.

Not only electron-withdrawing but also electron-donating groups could diminish the antibacterial activities unless a 2′-hydroxy was present in the 2-aryl substituent of the 4,5-dihydro-thiazole analogues. These results implied that the intramolecular hydrogen bond between the 2′-hydroxy and the nitrogen-atom of 4,5-dihydrothiazole is very important for the antibacterial activities of these compounds.

According to the fatty acid exposure experiments at high concentrations of fatty acid (1000 ppm), when short chain acids were added in culture medium, the bacteria grew normally; inhibition zones become small while long chain acids were added in it. The overall trend is consistent with the results of yanglingmycin, whereby by decreasing the concentration of fatty acids added, the inhibition zone gradually increased. This implied that these antibacterial compounds influence fatty acid synthesis of the tested bacteria. Scanning electron microscopy results showed that the bacterial cell walls showed depressions, leading to cell content leakage. Compound **7h** may damage cell membranes, leading to altered cell metabolism and the finally cell lysis and death. The antibacterial mechanism is similar to that of yanglingmycin.
